# Exploring the beliefs and perceptions of spending time in nature among U.S. youth

**DOI:** 10.1186/s12889-021-11622-x

**Published:** 2021-08-23

**Authors:** Astrid N. Zamora, Marika E. Waselewski, Abby J. Frank, Jack R. Nawrocki, Aspen R. Hanson, Tammy Chang

**Affiliations:** 1 Department of Nutritional Sciences, University of Michigan School of Public Health, Ann Arbor, MI USA; 2 Department of Family Medicine, University of Michigan, 2800 Plymouth Road, Building 14- Room G128, Ann Arbor, MI USA; 3 Community High School, Ann Arbor, MI USA; 4 College of Literature, Science, and The Arts, University of Michigan, Ann Arbor, MI USA; 5 College of Engineering, University of Michigan, Ann Arbor, MI USA; 6 Institute for Healthcare Policy and Innovation, University of Michigan, Ann Arbor, MI USA

**Keywords:** Youth, Nature, Adolescent, Mental health, Physical health

## Abstract

**Purpose:**

The prevalence of poor mental health continues to rise among youth; however, large-scale interventions to improve mental and physical health remain a public health challenge. Time spent in nature is associated with improved health among youth. This study aimed to assess youth experiences with nature and the self-perceived impact on their mental and physical health among a nationwide sample of US youth.

**Methods:**

In September 2020, five open-ended questions that aimed to assess perceptions regarding nature were posed to 1174 MyVoice youth, aged 14–24 years. Qualitative responses were analyzed using thematic analysis, and data were summarized using descriptive statistics.

**Results:**

The mean (SD) age of the 994 respondents (RR = 84.7%) was 18.9 (2.7) years; 47.4% were female, and 57.4% Non-Hispanic White. Among youth, many felt that spending time in nature positively impacted their mental health, with 51.6% mentioning that it made them “feel calm when I am out in nature”; 22.1% said that it relieved stress or “reduces my anxiety,” and 17.1% felt that being in nature positively impacted their physical health and “makes me feel more active and in shape.” However, 7.0% said it negatively impacted their health, such as “It makes me feel isolated.” Most youth (87.8%) want to spend more time in nature, with 22% mentioning barriers (i.e., busy schedules, built environment, and COVID-19) impeding them from doing so.

**Conclusions:**

Youth in our sample generally report feeling physically and mentally better when spending time in nature and want to spend more time in nature. Public health policies and practices that eliminate barriers and actively support time spent outside may be a feasible and acceptable practice to promote overall well-being among youth.

**Supplementary Information:**

The online version contains supplementary material available at 10.1186/s12889-021-11622-x.

## Introduction

The mental health of youth, defined as adolescents and young adults between the ages 15*–*24 years [1], is a growing public health concern [2]. In the United States (US), suicide is the second leading cause of death among those 15–24 years of age, with approximately one in five youth between the ages of 9–17 experiencing at least one or more mental health disorders [3]. Approximately 50% of all lifetime mental illnesses develop by age 14 and 75% develop by age 24 [4]. Mental health issues are not only prevalent in the US, but are also costly. The US spends more money on mental health services than any other country per capita [5]. Despite this investment, over 1 in 3 adolescents between the ages of 11*–*17 lack access to mental health services [6], demonstrating the need for accessible, affordable, and sustainable large-scale interventions.

In conjunction with risk for poor mental health, adolescence is a critical life-stage marked by various physiological changes (i.e., hormonal fluctuations) and lifestyle and behavioral changes that place youth at an increased risk of experiencing poor physical health outcomes [7, 8]. Many of these lifestyle and behavioral changes have been classified as social and environmental determinants associated with adverse mental and physical health among maturing adolescents. Determinants, such as academic-related time pressures [9], social media use [10–12], inadequate nutritional intake [13], smoking and alcohol use [14], begin to affect mental and physical health during adolescence but have the potential to persist into adulthood [15].

To promote health among youth, traditional interventions seek to address youth mental and physical health through cognitive behavioral therapy, participating in physical activity, dietary changes, and improving sleep hygiene, such as not using technology before bed [14]. Despite efforts to promote youth interventions, resource allocation for youth mental health remains limited [2]. One specific intervention that has shown promise in promoting mental health is time spent in nature [16, 17]. Multiple studies have found positive associations between children spending time in nature and mental health [16, 17].

Although studies are limited among youth, a systematic review among adults residing in mostly Western countries found strong positive associations between blue space (i.e., outdoor environments that feature water and are accessible either proximally or distally/virtually) and mental and physical health [18]. Potential mediators underlying associations between blue space and mental health include exposure to fewer noise and air pollutants in areas with more blue space, as well as reduced noise annoyance and masking of traffic noise via pleasant water sounds [19]. Similarly, greenspace (i.e., overall vegetation level and green spaces, such as parks and gardens) was linked to better mental health via indirect pathways, such as lower annoyance, among a large sample of Bulgarian college students [19]. Further, a recent study among 16–25-year-olds in Scotland found associations between exposure to greenspace and increasing physical activity, decreasing sedentary behavior and providing opportunities for restoration [20].

However, data on the perceived impact that nature has on the mental and physical health of youth remains inconsistent [15]. In one study of minority youth exposed to a 13-week environmental sustainability education program with a nature contact component, physical activity scores pre-intervention were not significantly different from scores post-intervention [21]. Similarly, a separate study among 15*–*25-year-old youth from Bulgaria found no association between greenspace and perceived mental health [22]. Contrarily, a national cross-sectional study among Canadian adolescents demonstrated the importance of adolescent engagement with nature as protective for their psychological well-being [23]. These discrepancies suggest a need for more research focused on assessing the impact between time in nature and health from the youth perspective.

Youth today spend less time in nature than the previous generations before them [24–26]. Studies indicate that as youth grow older, they begin to spend less time in nature and spend more time indoors [27]. Although it is unclear why youth are spending less time in nature, increased exposure and uptake of technology and electronic media may be associated with the decline in nature-based outdoor time among youth [28]. However, limited knowledge exists on youths’ beliefs regarding the amount of time they spend in nature and the potential barriers youth face to spending time outdoors. Understanding youth perceptions regarding the impact that spending time in nature has on their health is imperative in informing community-level policies and interventions that aim to support youth mental and physical health. Therefore, this study utilized a nationwide sample to describe the perceptions and experiences of youth related to the impact of nature on their mental and physical health.

## Methods

Data from answers given to five open-ended questions were obtained from the MyVoice cohort. MyVoice is an ongoing longitudinal national text message poll that seeks to understand youth opinions on salient issues related to health and policy. Participants are a diverse sample of over 1000 youth aged 14*–*24 years; details on the cohort’s demographics and study procedures have been previously described elsewhere [29]. This study was approved by the University of Michigan Institutional Review Board with a waiver of parental consent for minors.

**Fig. 1:**
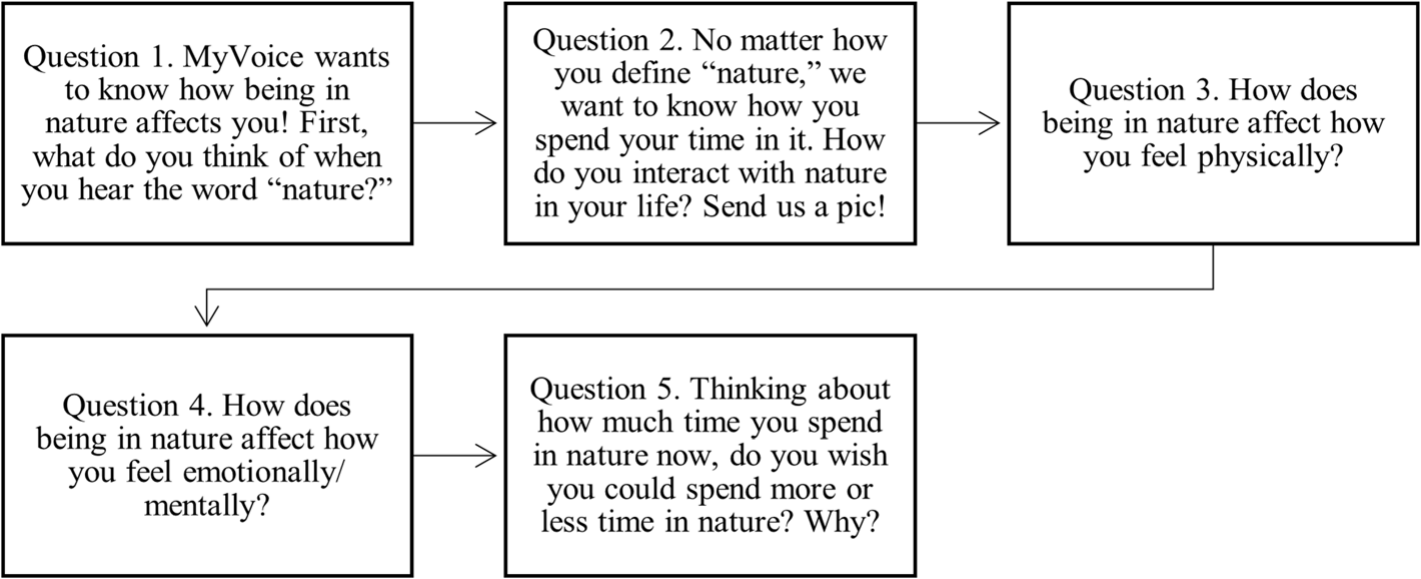
Nature question set sent to MyVoice participants on September 11, 2020

Questions were iteratively developed by a research team of youth, physicians, and mixed-methods research experts and were then sent to 1174 MyVoice participants on September 11, 2020. Figure 1 demonstrates the questions that were sent to participants to assess beliefs surrounding the amount of time spent in nature, as well as potential health impacts of spending time in nature:

The four open-ended text-based questions were included in this study. Qualitative data obtained from participant responses to each question were analyzed based on an inductive thematic analysis approach [30]. Responses to each question were reviewed independently by two investigators and major topics and ideas were identified and grouped. Two investigators then used discussion to condense their two lists into one. This list of major ideas was used to develop a comprehensive codebook that encompassed all of the responses. The codebook (Supplemental Table 1) defines all codes (i.e., word or phrase that reflected an opinion, thought, or feeling in response to the questions posed) that were identified. A written definition and examples for each code were also provided for clarity and to promote accuracy in coding. Specifically, for each question we performed a data-led approach with themes being determined inductively rather than relying on preconceived themes [31]. Two investigators then independently assigned codes to each text message response. Several codes could be applied within one response. Codes that were repeated in different questions by the same respondent were only counted once. Discrepancies between investigators were identified and discussed amongst the larger research team to reach a consensus on all applied codes. After completing the thematic analysis, we used SAS 9.4 software (Cary, NC, USA) to obtain percentages and frequencies for each of the codes identified in each study question.

## Results

Among this nationwide sample of 1174 MyVoice participants, 994 responded to at least one question (response rate = 84.7%) and were included in the present study. The mean (SD) age was 18.9 (2.7) years, with participants ranging from 14 to 24 years. Among the sample, 47.4% of the sample self-identified as female. More than half of the sample (57.4%) identified as Non-Hispanic White, and 39.7% of youth reported having less than a high school education (Table 1).

**Table 1:** Sociodemographic characteristics of respondents (*n* = 994)

Self-reported characteristics	n (%)
Age (years), mean (SD)	18.9 (2.7)
Age range	14–24
Gender	
Male	440 (44.3)
Female	471 (47.4)
Other Gender^a^	82 (8.3)
Race/Ethnicity	
Non-Hispanic White	569 (57.4)
Non-Hispanic Black	81 (8.2)
Hispanic	129 (13.0)
Non-Hispanic Other	213 (21.5)
Participant education level	
Less than high school^b^	394 (39.7)
High school grad	146 (14.7)
Some college or tech school	291 (29.3)
Associate’s or tech grad	33 (3.3)
Bachelor’s degree or higher	129 (13.0)
Region	
Midwest	336 (32.9)
Northeast	162 (16.3)
South	282 (28.5)
West	211 (21.3)
Eligible for free or reduced school lunch	
Yes	361 (36.6)
No	624 (63.4)

^a^Includes participants that self-identified as transgender, nonbinary or other

^b^Includes participants still in high school

When asked what youth think about when they hear the word “nature,” central themes that emerged from responses included: trees or woods (“I think of the outside like woods and water when I hear nature” or “I think of being deep in the woods near all of wildlife”; 45.1%); being outdoors (“Things that grow outdoors. Something grown from the Earth or made from things outside” or “Spending time outside with my family”; 35.5%); and green-spaces (“I think of plants and greenery” or “Green scenary”; 21.7%). Table 2 presents the major themes, frequencies, and representative quotes that emerged from youth responses to each question posed.

**Table 2:** Questions, codes, and representative quotes based on participant responses

Question, Code	n (%)^a^	Representative quote
**What do you think of when you hear the word “nature?” (*n*** **= 973)**		
Trees/woods	439 (45.1)	*“Tree, forest, flower... anything not man-made”* *“I think of being deep in the woods near all of wildlife”*
Outdoors	345 (35.5)	*“Spending time outside with family”* *“I think of riding my bike and being outside in general”*
Green-space/gardens	211 (21.7)	*“I think of green spaces with lots of plants”* *“Green scenery, flowers, animals, fresh air”*
Animals/bugs	168 (17.3)	*“Animals and the world”* *“Just being outside with the plants and animals and bugs.”*
Peaceful/beauty	152 (15.6)	*“I think of peace and serenity whenever I hear the word nature”* *“Calmness, being surrounded by something beautiful”*
Environment	145 (14.9)	*“I think of the earth!”* *“Nature is something that is from our environment that is not man-made”*
**How does being in nature affect how you feel physically? How does being in nature affect how you feel emotionally/mentally? (*n*** **= 885)**		
At peace/calm	457 (51.6)	*“I always feel calm when I am out in nature”* *“It helps me feel more balanced and connected, and more at peace with the world around me”*
Generally better	276 (31.2)	*“It makes me feel generally better, I have more energy and I feel like I can breathe easier”* *“I feel physically, emotionally, and mentally better after being in nature”*
Happier	268 (30.3)	*“Happier and more mindful of my emotions”* *“Nature greatly improve my emotions and mentality as it makes me happy”*
Refreshed/restored	261 (29.5)	*“It makes me feel refreshed. Being in nature is sort of a reset”* *“I feel mentally restored and energized!”*
Relieves or reduces stress and/or anxiety	196 (22.1)	*“Relieves a lot of stress and relaxes me”* *Nature helps me mentally. Gets rid of stress.*
Healthy/fit	151 (17.1)	*“It makes me feel more active and in shape”* *“It helps me feel like I am in better shape, healthier, and more connected”*
Negative	62 (7.0)	*“It makes me feel isolated”* *“I may feel anxious and may become more quiet or on edge”*
No Effect	63 (7.1)	*“Nature doesn’t have much of an effect on me, I can be relaxed in nature or stressed in nature”* *“I never really paid attention to how it affects me in this way”*
**Thinking about how much time you spend in nature now, do you wish you could spend more or less time in nature? Why? (*n*** **= 852)**		
More time	748 (87.8)	
*Experience barriers to time in nature*	177 (20.8)	*“I wish I could spend more time in nature to fully take advantage of it and enjoy it instead of being inside stuck at a computer doing online school for 8h hours a day”* *“I wish I could spend more time in nature. I live in the city so it can be hard to get out to go into the woods and hike”*
*Supports mental health*	149 (19.9)	*“Yes, I wish I could spend more time in nature so I could just relax and clear out my mind from these rough current times”* *“More time because it helps my mental health positively”*
*Feels good*	110 (14.7)	*“I wish I could spend more time in nature because it makes me feel good”* *“More because it is healthy and makes me feel good”*
*It is beautiful/ peaceful*	90 (12.0)	*“I wish I spent more time in nature, especially around sunset/sunrise because it is so beautiful”* *“More because I love how beautiful and relaxing it is”*
Same amount of time	71 (8.3)	
*Already spend enough*	7 (9.9)	*“Not really. I think I’m at a good spot right now because I don’t spend too much time but I also don’t spend too little time”*
Less time	29 (3.4)	
*Not into nature*	15 (51.7)	*“Less time. As little time as possible. I’d rather explore parking lots or alleyways”* *“Less time because I’m not that involved with it and I could be doing better things”*

^a^Codes are not mutually exclusive or representative of all codes applied and may therefore not total 100%

In response to how being in nature affected youth physically and mentally, more than half of all youth said that it made them feel at peace or calm (“Overall positive. Calms me down and is very good for a break from society” or “I always feel calm when I am out in nature”; 51.6%). Youth also commonly noted that being in nature made them feel generally better (“Generally I feel better about my life as a whole” or ““I feel physically, emotionally, and mentally better after being in nature”; 31.2%), and that it made them happier (“It makes me feel happy, and helps to clear my head” or “Nature greatly improve my emotions and mentality as it makes me happy”; 30.3%). Other themes included youth feeling refreshed or restored (“I feel mentally restored and energized” or “It makes me feel refreshed. Being in nature is sort of a reset”; 29.5%) and less stressed or anxious (“Nature helps me mentally. Gets rid of stress” or “Relieves a lot of stress and relaxes me”; 22.1%). However, a small percentage of youth felt that spending time in nature negatively impacted their mental or physical health (“A lot of people say it soothes them but nature doesn’t soothe me. It makes me feel unwelcome. I hate the bugs mostly” or “It makes me feel isolated”; 7.0%).

When asked to consider the current amount of time they spend in nature and if they would like to spend more or less time in nature, a majority of youth (87.7%) said they wanted to spend more time in nature (Table 2). One reason why youth wanted to spend more time in nature was because it supports their mental health (“I wish I could spend more. It would ease my stress level” or “More time because it helps my mental health positively”; 19.9%). Youth also mentioned that it just makes them feel good (“More time, because it makes me feel good”; 14.7%); and because nature is beautiful (“More time because nature is such a beautiful mystical place” or “More because I love how beautiful and relaxing it is”; 12.0%). However, a small group of youth did indicate they would like to spend the same amount of time in nature (“I already spend quite a bit of time in nature, but I’d keep it the same” or “Not really. I think I’m at a good spot right now because I don’t spend too much time but I also don’t spend too little time”; 8.3%). An even smaller percentage noted wanting to spend less time in nature (“Maybe less. I don’t like it much” or “Less time. As little as possible. I’d rather expore parking lots and alleyways”; 3.4%).

Among youth that reported wanting to spend more time in nature (87.7%), a total of 177 (20.8%) described barriers that keep them from spending more time in nature (Table 2). Most commonly noted (97; 11.4%) was that school and work responsibilities were a significant barrier to spending more time in nature (“I wish I could spend more time in nature, especially because my activities with remote learning are increasingly inside now and I don’t think it’s a good thing to be inside for a long time”). Others (49, 5.8%) felt that the built environment was an impediment to spending more time in nature (“I wish I could spend more time in nature because I live in an urban area with little green space”). Finally, 40 youth mentioned that current fear or restrictions related to COVID-19 were barriers to spending more time in nature (“I wish I could spend a bit more time, but with covid, it’s scary”; 4.7%).

## Discussion

In seeking to understand youths’ experiences with nature and its impact on their mental and physical health, we found that most youth perceive nature as a space that exists outside, such as trees/woods, general outdoors, and green-spaces. Youth in our survey also commonly perceived time spent in nature as positively impacting their mental and physical health. As a result, many youth (87.8%) indicated wanting to spend more time in nature but noted that various barriers in their lives impacted their time and access.

Little is known about what youth perceive as nature or being in nature. While previous work has focused on understanding how children define and perceive nature [32, 33], few studies have documented how adolescents and young people define and perceive nature [34]. Our results suggest that youth do not have a narrow or formal definition of what nature means and instead broadly define nature as a space that exists outdoors. This understanding of how youth define nature may have public health implications, given that youth may not need to travel far or spend much money to access nature. Though some youth live in unsafe neighborhoods with limited green space, spending time in nature (i.e., outside) for many youth is free and accessible, but underused [35]. However, we also found that nearly half of all participants think of trees and/or woods when they hear the word nature. Given that access to trees and/or woods is not equitably distributed or accessible to all Americans [36], our findings may shed light on the importance of creating more accessible natural spaces with trees and woods within urban settings or making these spaces accessible by public transit to urban youth within the US.

A large percentage of youth in our sample found nature to impact their mental health positively. Our findings add to the body of literature by providing supporting evidence demonstrating that exposure to greenspace is associated with lower perceived stress [37, 38]. Youth reported that being in nature relieves or reduces stress or anxiety and helps them feel at peace or calm. These findings align with previous research among European youth, which found that nature provides relief and calm among youth [34]. Although our nationwide sample of US youth may be different from European youth, and spatial patterning of city-rural gradients in Europe likely differ from that of the US, similar findings may signify that the impact of nature holds across diverse youth populations. Moreover, our findings align with previous research among US youth from a Midwestern state demonstrating an inverse relationship between green spaces and levels of perceived stress [39].

Many youth also reported that being in nature positively impacted their physical health, which aligns with research that demonstrated the relationship between nature and physical health [16]. To illustrate, a study among adolescents found that the prevalence of overweight dropped by 14% with each increase in hours spent outdoors [40]. Health promotion guidelines universally recommend physical activity, which often occurs outdoors. Within the past decade primary care providers have “prescribed” nature to patients, namely children, as an alternative to medication [41]. However, without explicitly recommending time spent in nature, the opportunity for the additional benefits of being outdoors is lost. Of note, a small subset of youth in our study reported that spending time in nature had a negative impact on their physical health. Most of these responses were related to youth being physically affected by elements, such as heat and insects. Although we did not identify relevant literature supporting the idea that exposure to nature has a negative impact on physical health, a previous study among a sample of minority youth in the US exposed to nature found that exposure had no significant effect on physical health outcomes [21].

Leisure-time physical activity increases during middle childhood and significantly decreases across adolescence [42]. For example, only 2% of US high schools provide daily outdoor physical education throughout the academic school year [43]. Youth in our study reported wanting to spend more time in nature but many could not due to barriers such as demanding schedules, mostly due to academic and social pressures, the built environment, and fears and restrictions related to the current COVID-19 pandemic. To our knowledge, our study is among the first to report the barriers youth perceive to spending time in nature. Existing literature has focused on barriers to spending time in nature among children from the perspective of their guardians and caretakers, which have noted that the most common barriers that discourage time spent in nature are safety concerns and lack of access to outdoor spaces [35]. One difference, was that participants in our sample reported fears and restrictions due to the COVID-19 pandemic, which may be a unique barrier specific to the current period of time. Similar to the comparison study, participants in our sample reported that the built environment was a barrier to spending more time in nature [35]. These findings support the need for a multi-sectoral approach to promote outdoor time among youth, including by schools, especially amid virtual learning environments, which many of our youth noted as creating barriers to spending more time outdoors. Ensuring safe outdoor spaces are accessible and attractive to young people will require support from many stakeholders, including those that support, fund, build, protect, maintain, and use outdoor spaces.

Our study has several limitations. Targeted recruitment was performed to maximize participant representativeness [29]; however, our sample is not nationally representative and may be subject to selection bias through social media recruitment. Though our study had a high response rate, non-respondents may have had little interest or exposure to the topic of nature, leading to bias in our results. Another limitation of the study was that participants responded to the question set during the COVID-19 pandemic, which was characteristic of lockdown restrictions which may have influenced participants responses related to spending time in nature. Lastly, while open-ended text message questions allow participants to respond in their own words, we could not probe further for clarifications as is possible during in-person interviews.

In summary, youth perceive nature as having a positive impact on their mental and physical health, and most would like to spend more time in nature. However, some youth face barriers to time spent in nature, including demanding school schedules, the built environment, and COVID restrictions. Findings from this study may inform public health interventions and policies that aim to support youth health; in particular, explicitly enabling and encouraging youth to spend more time outside in nature may serve as a simple public health strategy to support US youth’s overall health and well-being.

## Supplementary Information

**Additional file 1: Supplemental Table 1**. Codebook including codes, definitions, and example responses.

## Data Availability

The datasets used and/or analysed during the current study are available from the corresponding author on reasonable request.
