# Supplementary material for: Exploring the beliefs and perceptions of spending time in nature among U.S. youth
Source: BMC Public Health. 2021 Aug 23;21:1586. doi: 10.1186/s12889-021-11622-x (PMC8381719; doi:10.1186/s12889-021-11622-x)
Supplement: Supplementary file 1 — Additional file 1: Supplemental Table 1. Codebook including codes, definitions, and example responses. [file 12889_2021_11622_MOESM1_ESM.docx]

**Supplemental Table 1**. Codebook including codes, definitions, and example responses

| Code | Definition | Example response |
| --- | --- | --- |
| **What do you think of when you hear the word “nature?”** | | |
| Trees/woods | A response that indicates the respondent correlates nature with trees, woods, or forests. | “I think of a forest or grove, anywhere with a lot of trees.” |
| Outdoors | A response that indicates the respondent correlates nature with the outdoors or being outside. | “The great outdoors” |
| Green-space/gardens | A response that indicates the respondent correlates nature with greenery, flowers, grass, or plants. | “I think of plants and greenery.” |
| Animals/bugs | A response that indicates the respondent correlates nature with wild animals, pets, or bugs. | “I think of birds chirping and animals grazing.” |
| Peaceful/beauty | A response that indicates the respondent correlates nature with a feeling of peace, happiness, relaxation, freedom, escaping, quietness, or beauty. | “I think of beauty, serenity, and peace.” or “when you find your inner peace or a place where you can relax” |
| Environment | A response that indicates the respondent correlates nature with the environment, universe, naturalness, wilderness, or openness. | “I think of vast open spaces.”  “Nature to me means the environment.” |
| Water | A response that indicates the respondent correlates nature with water and bodies of water (lakes, oceans, rivers, etc.). | “Rivers, lakes, and oceans” |
| Activities | A response that indicates the respondent correlates nature with activities such as sports, camping, exploring, hiking, walking, etc. | “Taking hikes, camping, stargazing” |
| Sky/weather | A response that indicates the respondent correlates nature with sky, weather, or fresh air. | “When I hear the word nature, I think of rain and severe weather.” |
| Mountains | A response that indicates the respondent correlates nature with mountains. | “I think of being surrounded by mountains.” |
| Parks | A response that indicates the respondent correlates nature with parks (national, public, state, etc.). | “I picture a place like a national park.” |
| Family/friends | A response that indicates the respondent correlates nature with family and friends. | “Spending times outside with family” |
| **How does being in nature affect how you feel physically? How does being in nature affect how you feel emotionally/mentally?** | | |
| At peace/calm | A response that indicates that nature makes the respondent feel calmer, relaxed, or at ease. | “Me being in nature makes me feel relaxed and calm.” |
| Generally better | A response that indicates that nature makes the respondent feel better emotionally and healthier. | “I feel so much better in every way.” |
| Happier | A response that indicates that nature makes the respondent feel happier or improves their mood. | “I feel happier.” |
| Refreshed/restored | A response that indicates that nature makes the respondent feel more refreshed, restored, connection to something bigger, more energetic, or freer. | “Being in nature makes me feel more refreshed…”  “I feel energized and ready.” |
| Relieves or reduces stress and/or anxiety | A response that indicates that nature makes the respondent feel relieved of stress and/or anxiety. | “It makes me feel relaxed and allows me to let loose any problems or stress I am feeling.” |
| Healthy/fit | A response that indicates that nature makes the respondent feel healthier, stronger, more fit, or more adventurous. | “It makes me feel physically healthy.”  “I use hiking as a main form of exercise , so I spend a lot of time outside and its nice to get out and walk.” |
| Negative feeling | A response that indicates nature makes the respondent feel uneasy, scared, uncomfortable, anxious, or annoyed. | “I was scared and overwhelmed honestly...”  “...Depending on where I am I can be a bit anxious.” |
| No effect | A response that indicates that nature makes the respondent feel no effect or change. | “It does not particularly affect me physically” |
| Allergies | A response that indicates that nature makes the respondent experience allergies. | “Considering my severe allergies not good.” |
| Tired/Worse | A response that indicates that nature makes the respondent feel tired or worse. | “I felt more tired and sluggish.” |
| **Thinking about how much time you spend in nature now, do you wish you could spend more or less time in nature? Why?** | | |
| More time | A response that indicates they would like to spend more time in nature than they do now. | “I wish for so much more” |
| Same amount of time | A response that indicates they would like to spend a similar amount of time in nature as they do now. | “I’m ok with how much time I spend in nature” |
| Less time | A response that indicates they would like to spend less time in nature than they do now. | “Less. I only occasionally go on hikes and stuff, and that’s too often” |
| Experience barriers to time in nature | A response that indicates they would like to spend **more time** in nature but are *unable to* because of busy schedules (work, school, etc.), the built environment where they live, or COVID-19 and quarantining | “…I live in a city where there’s barely any nature”  “I definitely wish I could spend a lot more time in nature, I just can’t because school work and everything” |
| Supports mental health | A response that indicates they would like to spend **more time** in nature because it supports mental health by stress and anxiety reduction, decompressing, etc. | “I wish I could spend more time in nature because of its calming and euphoric effects” |
| Feels good | A response that indicates they would like to spend **more time** in nature because it generally feels good | “More time, because it makes me feel good” |
| It is beautiful/peaceful | A response that indicates they would like to spend **more time** in nature because they find it beautiful, peaceful, serene, or soothing | “It's beautiful and much more enjoyable then being inside all day” |
| Like exploration | A response that indicates they would like to spend **more time** in nature because they love exploring, seeing new things, traveling, or generally love nature | “More! I grew up in the woods and love exploring and being in them” |
| Supports physical health | A response that indicates they would like to spend **more time** in nature because it supports physical health or health in general | “I wish I could spend way more time in nature because it makes me feel healthier” |
| Already spend enough time | A response that indicates they would like to spend a **similar amount** of time in nature because they are content with the amount of time they spend in it or already spend a lot of time in it. | “The same. I’m out a good amount of time” |
| Not into nature | A response that indicates they would like to spend **less or similar amount** of time in nature because they don’t really enjoy being in nature and don’t need to spend time in it. | “I’m not interested in nature at all. I am terrified of bugs!” |
| Content inside | A response that indicates they would like to spend **less or similar amount** of time in nature because they feel content to spend their time indoors. | “Honestly, I *could* spend more time outside, I just choose not to; as I said, I'm pretty happy indoors” |
